# Case Report: Novel *SLC9A6* Splicing Variant in a Chinese Boy With Christianson Syndrome With Electrical Status Epilepticus During Sleep

**DOI:** 10.3389/fneur.2021.796283

**Published:** 2022-01-14

**Authors:** Xiaorui Liu, Lingling Xie, Zhixu Fang, Li Jiang

**Affiliations:** Ministry of Education Key Laboratory of Child Development and Disorders, Chongqing Key Laboratory of Pediatrics, Department of Neurology, National Clinical Research Center for Child Health and Disorders, Children's Hospital of Chongqing Medical University, Chongqing, China

**Keywords:** *SLC9A6*, Christianson syndrome, electrical status epilepticus during sleep, splicing mutation, reverse transcription-polymerase chain reaction

## Abstract

We investigated the existence and potential pathogenicity of a *SLC9A6* splicing variant in a Chinese boy with Christianson Syndrome (CS), which was reported for the first time in China. Trio whole-exome sequencing (WES) was performed in the proband and his parents. Multiple computer prediction tools were used to evaluate the pathogenicity of the variant, and reverse transcription-polymerase chain reaction (RT-PCR) analysis and cDNA sequencing were performed to verify the RNA splicing results. The patient presented with characteristic features of CS: global developmental delay, seizures, absent speech, truncal ataxia, microcephaly, ophthalmoplegia, smiling face and hyperkinesis with electrical status epilepticus during sleep (ESES) detected in an electroencephalogram (EEG). A *SLC9A6* splicing variant was identified by WES and complete skipping of exon 10 was confirmed by RT-PCR. This resulted in altered gene function and was predicted to be pathogenic. ESES observed early in the disease course is considered to be a significant feature of CS with the *SLC9A6* variant. Combined genetic analysis at both the DNA and RNA levels is necessary to confirm the pathogenicity of this variant and its role in the clinical diagnosis of CS.

## Introduction

The *SLC9A6* gene(chrX:135098802), located on Xq26.3, encodes isoform 6 of the Na^+^/H^+^ exchanger superfamily (NHE6). NHE6 exchanges luminal H^+^ in early and recycling endosomes, thus contributing to substance transport and receptor recycling, which are essential for axonal growth, branching, synaptic maturation and neural plasticity (1). Christianson syndrome (CS) has been associated with a *SLC9A6* variant and is characterized by moderate to severe global developmental delay, epilepsy, absent or impaired speech, truncal ataxia, ophthalmoplegia, acquired microcephaly (2) and reduced life expectancy (3). The clinical features of CS overlap with those of Angelman syndrome (AS) (4), making it hard to identify in clinical practice.

In this study, we identified a maternally-inherited *SLC9A6* splicing variant in a Chinese boy who presented with global developmental delay, epilepsy and microcephaly. The whole-exome sequencing results were further verified by reverse transcription-polymerase chain reaction (RT-PCR) and cDNA sequencing. Our findings indicate the pathogenicity of this *SLC9A6* splicing variant in CS.

## Materials and Methods

### Patients and Samples

The proband was registered at the Department of Neurology of the Children's Hospital of Chongqing Medical University (China). Detailed neurological examinations were performed by at least two senior neurologists. Written informed consent was obtained from the participants, and the research project was approved by the Children's Hospital of Chongqing Medical University Ethics Committee. A normal sample was used as control.

### Whole-Exome Sequencing

Genomic DNA was extracted from the peripheral blood cells of the proband and his parents, as well as the normal control using a DNA extraction kit (Tiangen, Beijing, China). Whole exome DNA was captured using IDT The xGen Exome Research Panel v2.0 and subsequently sequenced on an Illumina NextSeq 500 system with 101-bp paired-end reads to screen for variants. After duplicated reads were removed from downstream analysis, clean reads were aligned to GRCh37/hg19 human reference genome using NovoAlign software. SNP and small insertion or deletion (InDel) variants were detected and identified using the Genome Analysis Toolkit (5) and then were annotated using ANNOVAR. Variants which fulfilled the following criteria were considered candidate genes: (a) variants that were absent or rare (allele frequency <0.01) in the 1,000 Genomes Project, Exome Aggregation Consortium (ExAC) or GnomAD databases; (b) variants that affected the amino-acid sequence, such as frameshift, and splice site variants. The pathogenicity of the identified variants was then predicted using multiple algorithms prediction tools, such as PolyPhen-2, Mutation Taster, and Sorting Intolerant from Tolerant (SIFT), and classified according to the guidelines of American College of Medical Genetics and Genomics (ACMG) (6).

### Sanger Sequencing

Sanger sequencing was performed to validate the potentially pathogenic variations identified in the patient and his parents to determine the parental origin. The phenotypes of the proband and his parents were verified according to published articles (1, 3, 7–13) and OMIM database (OMIM:300243).

### RNA Splicing Analysis by RT-PCR

Multiple computer prediction tools (MaxEntScan, NNSPLICE, NetGene2, Alternative Splice Site Predictor, and FSPLICE) were used to evaluate the pathogenicity of variant. The RNA splicing results were verified by RT-PCR analysis and cDNA sequencing. Total RNA was extracted from the peripheral blood cells of the proband and normal control using an RNA extraction reagent kit (Tiangen, Beijing, China) and then converted into cDNA using the PrimeScript™ II 1st strand cDNA synthesis kit (Takara Dalian, China). Primers were designed to amplify the target fragment of the *SLC9A6* (forward: 5'-TACGGGAGTTCCAGTTGTTGG-3' and reverse: 5'-AGGGGTSSSTSTTGGCAGCTCTT-3'). After being isolated by agarose gel electrophoresis, the purified PCR products were sequenced by Sanger sequencing.

## Results

### Clinical Presentation

A Chinese boy was born at term with a birth weight of 2,950 g (25–50th centile) and head circumference of 32 cm (10–25th centile) without neonatal asphyxia at birth or aberrant family history. He developed complex febrile seizures at the age of 11 months, which manifested as a clustering of generalized tonic or tonic-clonic seizures (9 seizures within 2 days) lasting 15–60 s. Prior to the onset of seizures, the patient presented with developmental delay. He was admitted to our hospital at the age of 17 months, when his weight was 10.5 kg (25th centile), and head circumference was 42 cm (<1st centile), indicating microcephaly, with strabismus, narrow face, small mandible and frequent smiling. A café-au-lait spot (diameter 2.5 cm) was located on the lower left quadrant of the boy's abdomen. He could not stand independently or speak coherently. CS was diagnosed after *SLC9A6 pathogenic* variant identified by genetic analysis. An advanced clinical diagnosis was not given due to lack of sufficient cognition of this syndrome. Seizures were controlled for 3 months following treatment with valproate at 17 mg/(kg.d). Subsequently, afebrile seizure recurred almost once a month with the same type and duration as before. Valproate was increased to 45 mg/(kg.d) and Levetiracetam was included in the drug regimen when the patient was 1 year and 9 months old. At the last follow-up when the patient was 2 years and 10 months old, his seizures were controlled and this continued for more than 1 year although his electroencephalogram (EEG) remained abnormal. Rehabilitation training was performed but without adequate efficacy. The child had an ataxic gait, gradually developed hyperkinesis and was still unable to speak coherently.

### Electroencephalogram and Brain MRI Finding

**Figure 1:**
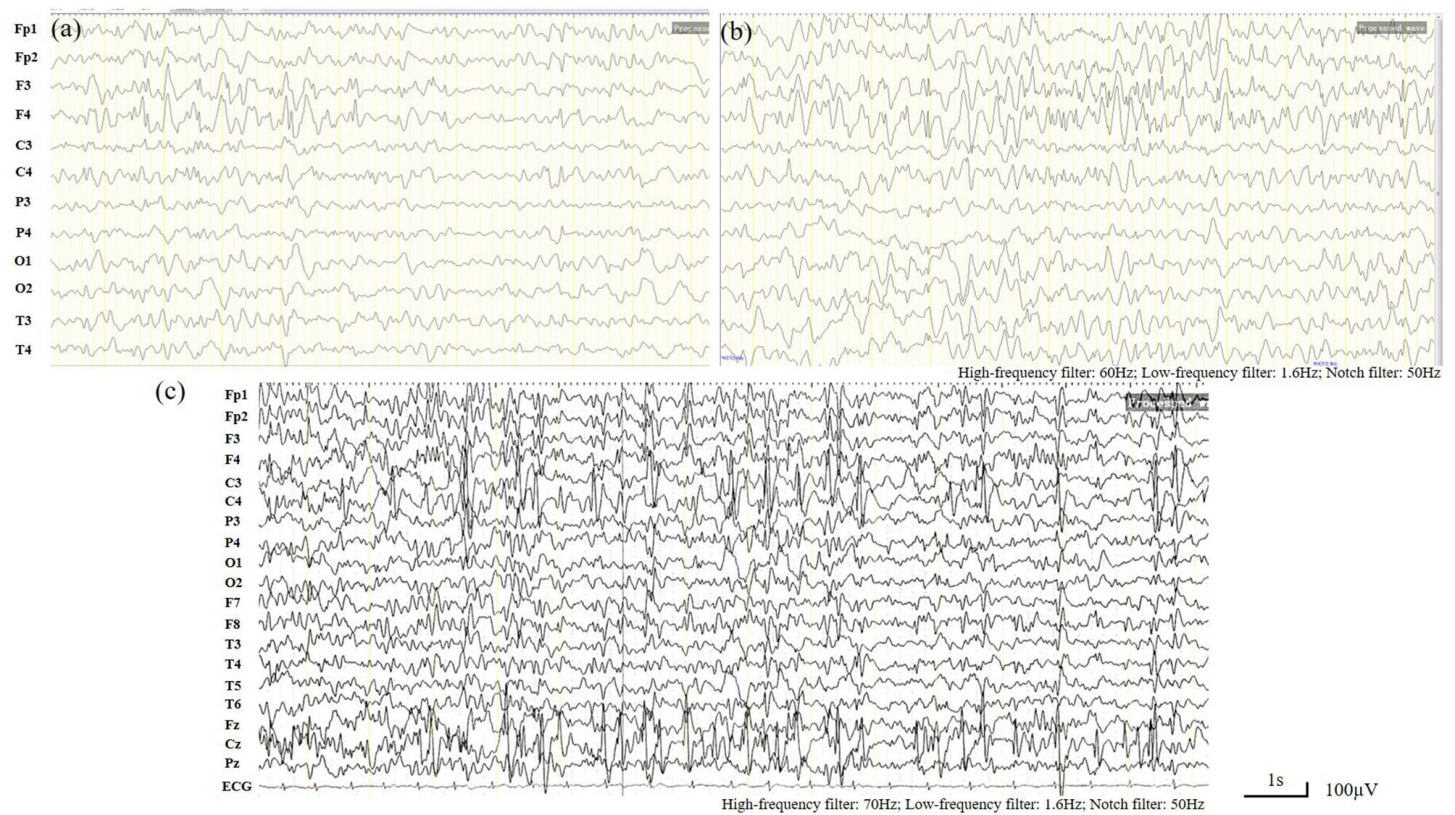
Electroencephalogram (EEG). **(a,b)** Routine sleep EEG tracing at the age of 11 months. Continuous spike-waves from the bilateral frontal regions, forming more than 80% of the sleep recording, suggesting an electrical status epilepticus during slow-wave sleep (ESES) like EEG finding. **(c)** Overnight sleep EEG tracing at the age of 2 years and 10 months. Almost continuous spike- waves predominantly over both frontal regions were occupying more than 80% of the sleep recording, which is consistent with ESES.

**Figure 2:**
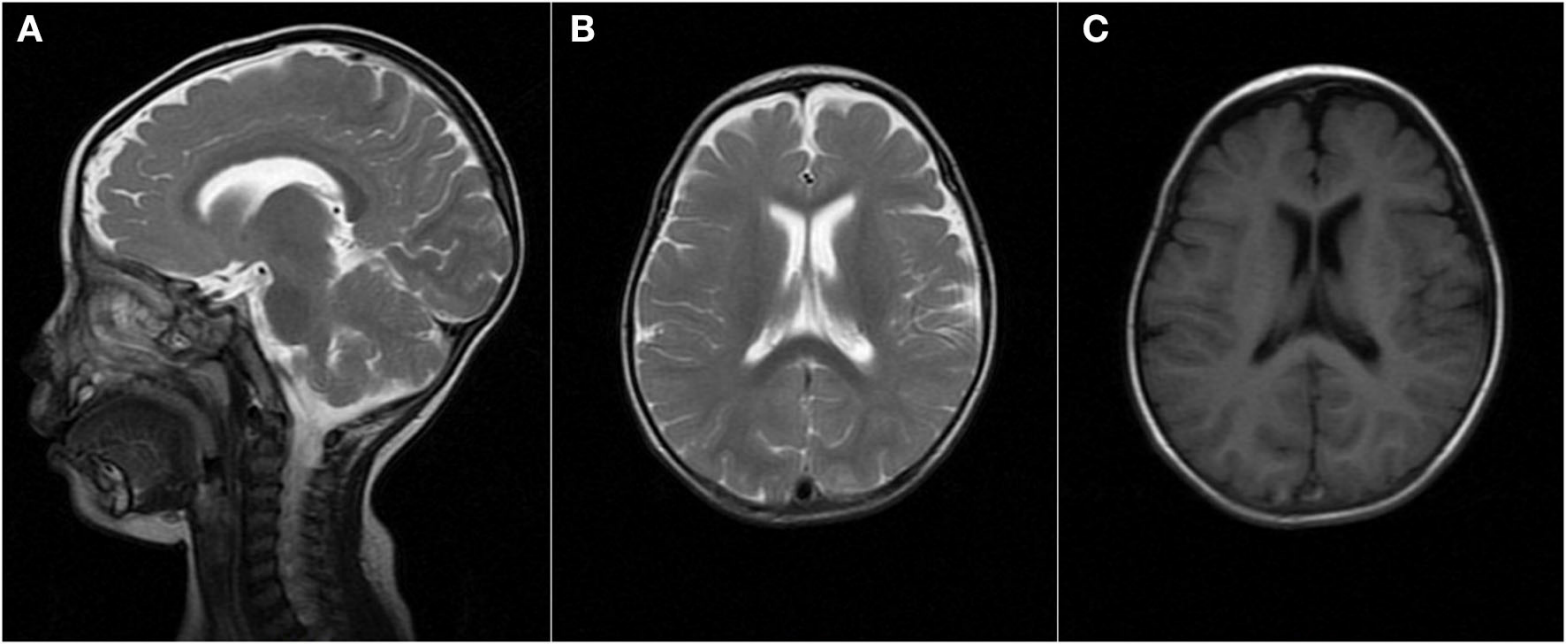
Brain MRI finding at the age of 11 months. MRI [**(A,B)** T2, **(C)** T1] demonstrated the widening of the extracerebral space at the bilateral temporal poles.

When the patient first experienced complex febrile seizures at the age of 11 months, the routine sleep EEG evaluation showed continuous spike-waves from the bilateral frontal regions, forming more than 80% of the sleep recording, which suggests the presence of electrical status epilepticus during slow wave sleep (ESES) (Figure 1). At that time, brain magnetic resonance imaging revealed widening of the extracerebral space at the bilateral temporal poles (Figure 2). ESES was still detected after the seizures were controlled for more than 1 year when he was 2 years and 10 months old and further confirmed during an overnight sleep EEG (Figure 1).

### Molecular Studies

Trio WES was performed on the patient and his parents, with written informed consent. A heterozygous, maternally-inherited *SLC9A6* splicing pathogenic variant [c.1237-2 (IVS9) A>G, NM_001042537] was identified and confirmed by Sanger sequencing. The variation was not present in the gnomAD, dbSNP, and ClinVar databases. At least three prediction tool algorithms predicted that this variant is associated with deleterious effects on the gene or gene products. These changes were classified as “pathogenic” (PVS1+PM2+PP3) according to ACMG criteria. His mother had the same variation bit in a heterozygous state and did not present any pathological clinical findings.

*In silico* analysis using multiple algorithms such as MaxEntScan, NNSPLICE, NetGene2, Alternative Splice Site Predictor, and FSPLICE predicted a strength reduction in the 3' acceptor site, which may disrupt the normal pre-RNA splicing.

**Figure 3:**
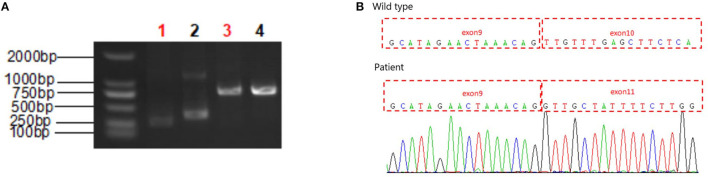
RT-PCR analysis of the proband. **(A)** RT-PCR electrophoresis products on agarose gel. Lane 1: SLC9A6 gene amplification in proband's sample, Lane 2: SLC9A6 gene amplification in normal sample, Lane 3: Reference gene amplification in proband's sample, Lane 4: Reference gene amplification in normal sample. SLC9A6 gene mRNA expression in peripheral blood cells. **(B)** Sequencing analysis. A skipped exon 10 showed in proband's cDNA compared to the sequencing analysis of a wild type sample.

To validate the RNA splicing prediction, we performed RT-PCR analysis and cDNA sequencing of the RNA extracted from the peripheral blood cells of proband and normal sample. Separation to of the PCR product by agarose gel electrophoresis confirmed expression of the *SLC9A6* gene transcript expressed in peripheral blood cells of the proband. PCR amplification and sequencing of the exons flanking the target loci confirmed abnormal splicing near the mutation site with complete skipping of exon 10 (Figure 3). This aberrant transcript resulted in a 38 amino-acid deletion (p.Leu381_Phe418del), and hence impaired the function of the NHE6 protein.

## Discussion

NHE6, encoded by *SLC9A6*, is predominantly located in early and recycling endosomes and operates as an alkalinizing mechanism that regulates luminal H^+^ levels. This process is essential for ligand-receptor complex dissociation and dephosphorylation (13), which are crucial for synaptic maturation and neural plasticity. NHE6 abnormalities can also affect cell viability (14), possibly due to disruption of the balance of apoptosis as a result of endosomal dysfunction and attenuation of tyrosine receptor kinase B (TrkB) signaling (8), although the mechanism remains to be elucidated. *SlC9A6* knockout mice presented an abnormal accumulation of GM2 ganglioside and unesterified cholesterol within late endosomes and lysosomes, with slow degeneration of neurons in the hippocampus, some areas of cerebral cortex and cerebellar Purkinje cells and a CS-like clinical phenotype (7). In addition to NHE6 loss-of-function mutations, which have been confirmed to be correlated to the pathophysiology of CS with *SLC9A6* variants, NHE6 gain-of-function mutations have also been reported to be associated with impairment of optimal recycling endosomal function, accounting for the pathophysiology of CS (12).

This is the first report of CS caused by a *SLC9A6* pathogenic variant in a patient in China. *SLC9A6* variants are correlated with various neurological diseases, including CS, autism spectrum disorders, schizophrenia and idiopathic Parkinson's disease (13). The correlation with CS has been well-established. CS follows a X-linked recessive inheritance pattern, with clinical features that overlap with those of AS in male patients presenting with moderate to severe global developmental delay, epilepsy, absent or impaired speech, truncal ataxia, ophthalmoplegia, acquired microcephaly, hyperkinesis, cerebellar atrophy and reduced life expectancy (Table 1) (1, 3, 7–13). Seizure onset usually occurs in late infancy. Tonic/tonic-clonic seizures are the most common types, although atonic seizures, myoclonic seizures and focal seizures have also been described (3). Phenotypes of Lennox-Gastaut syndrome are relatively common in CS patients, especially those with ESES (3). A case with spasms and hypsarrhythmia detected by EEG was also reported and classified as infantile spasms (14). There is very little information about the course of seizure development. However, there are some reports of spontaneous attenuation during adolescence (15), although moderate-to-severe intellectual disability and other neurologic abnormalities remain. Female carriers usually have a normal-to-mild phenotype with differences in penetrance and enhanced effects on progeny (16, 17). Our patient also presented with global developmental delay, generalized tonic/tonic-clonic seizures, absent speech, truncal ataxia, acquired microcephaly, ophthalmoplegia, smiling face and hyperkinesis, which is consistent with previous reports. His mother carried the same variation in a heterozygous state and presented with a normal phenotype.

**Table 1:** Differential characteristics between Christianson syndrome and Angelman syndrome.

	**Christianson syndrome**	**Angelman syndrome**
Gene	SLC9A6	Maternal gene UBE3A
		Chromosome 15q11.2-q13
Gender	Male	No gender preference
Seizure	Yes	Yes
Psychomotor delay	Yes	Yes
Speech deficits	Yes	Yes
Developmental regression	Motor regression	No
Intellectual disability	Always severe	Variable
Acquired microcephaly	Yes	Majority (more common in the deletion subtype)
Happy demeanor	Possible	Yes, often with hand-flapping movements
Facial features	Long thin face, quint, prominent jaw	Flat occiput, wide mouth, widely spaced teeth, protruding tongue, prognathism, hypopigmented
Strabismus	Yes	Possible
Ataxic gait	Yes	Yes and/or tremulous movement of the limbs
Hyperkinesis	Yes	Yes
Autistic features	Yes	Possible
Sleep disturbances	Possible	Yes
Weight gain	Poor	Poor in early childhood, normal or even obesity in young adulthood
Progressive cerebellar atrophy	Yes	No
ESES	Possible	No
Life span	shorter	normal

*ESES, electrical status epilepticus during sleep*.

Generalized slow spike-wave complexes as a predominantly interictal EEG feature of patients with *SLC9A6* variants and the presentation of ESES during early childhood (3 years 3 months−8 years) (3, 10, 15, 18, 19) was first described in 2014 (10). The characteristics of CS with ESES reported so far were summarized in Table 2. Patients with ESES are usually refractory to anti-epileptic drugs. Rohini et al. (19) reported resolution of ESES following felbamate treatment in a child with a *SLC9A6* variant. In our patient, who was much younger than those previously described, ESES-like EEG finding was present from the first episode of complex febrile seizures and was further confirmed without much attenuation after the seizures were controlled for more than 1 year. Thus, combined with the characteristic clinical presentation, ESES or ESES-like EEG finding is implicated as a valuable diagnostic indicator of the *SLC9A6* mutation.

**Table 2:** Clinical characteristics of 8 cases of CS with ESES.

**Cases**		**Gender**	**Age at epilepsy onset**	**Seizure type**	**Status**	**Seizure frequency**	**Resistance to AEDs**	**AEDs**	**Clinical features**	**Age at ESES**	**Age at ESES resolution**	**Development**	**Brain imaging**	**Genetic analysis**
1	Mathieu et al. (15)	Male	13 months	GTCS, myoclonic	NK	Yearly to free at 12 y	Yes	VPA, CLB, LEV, ESM, LTG	Delayed psychomotor development, no oral speech, ataxia gait, motor regression at 11 y, frequent smiling, microcephaly, low weight, dysmorphic features including long thin face, quint, prominent jaw	6 y	8 y	Moderate to severe ID	Slight enlargement of subarachnoid spaces, mostly in bitemporal regions, and a left temporal arachnoid cyst. (at 18 m)	40-Mb deletion in Xq26.3
2	Mathieu et al. (15)	Male	20 months	GTCS	Yes	Monthly	Yes	VPA, LEV, CLB^*^	Delayed psychomotor development, autistic features, no oral speech	4 y 10 m	NK	Moderate to severe ID	Normal (at 22 m)	c.1569G>A
3	Mathieu et al. (15)	Male	17 months	Partial seizures, GTCS	NK	NK	Yes	VPA, LTG, OXC, CLB^*^	Delayed psychomotor development, on oral speech, ataxia gait, underutilization of the right hand, sleep difficulties	4 y	NK	Moderate to severe ID	Normal (at 3 y)	c.1148G>A (p.Gly383Asp)
4	Zanni et al. (10)	Male	<2 years	TCS	NK	NK	Yes	NK	Delayed motor development, no oral speech, autistic features, microcephaly, severe hypotonia, ataxia gait, motor regression at 7 y	7 y	NK	Moderate to severe ID	Cerebellar vermin atrophy, cerebral and hippocampi atrophy (at 7 y)	c.1151-1G>A(IV10-1 G>A)
5	Coorg et al. (19)	Male	12 months	complex motor seizures, TCS	Yes	Yearly to free at 8 y (after initiation of felbamate)	No	CBZ, LEV, VPA, CLB, Felbamate	delayed global development, microcephaly, autistic features, sleep difficulty	8 y	NK	Moderate to severe ID	Normal (at 12 and 28 m)	c.1710G>A (p.Trp570^*^)
6	Ikeda et al. (3)	Male	17 months	GTCS, atonic seizures	NK	Daily	Yes	VPA, CLB, TPM, LTG, LEV, CBZ, Rufinamide	Delayed psychomotor development, microcephaly, no oral speech, ataxia gait, truncal hypotonia, hyperkinesis	7 y	NK	Severe ID	T2 hyperintensity and atrophy of the lower cerebellum (at 6 y)	c.477_481del (p.Ile160Leufs^*^5)
7	Gong et al. (18)	Male	1 year 11 months	Focal seizure, febrile GTCS, myoclonic seizures, atypical seizures	NK	Uncontrolled	Yes	VPA, LEV	Delayed psychomotor development, ataxia gait, on oral speech, hyperkinesis	3 y 3 m	NK	NK	Normal (age NK)	c.1178_1180del (p.394del)
8	Case	Male	11 months	Febrile GTCS, GTCS	No	Monthly to free at 1 year 9 months (after initiation of LEV)	No	VPA, LEV	Delayed psychomotor development, no oral speech, microcephaly, ataxia gait, hyperkinesis, frequent smiling, dysmorphic features including narrow face, strabismus, small mandible, café-au-lait spot.	ESES-like at 11 m, ESES diagnosed at 2 y 10 m	NK	Moderate to severe ID	Widening of the extracerebral space at the bilateral temporal poles (at 11 m)	c.1237-2 A>G (IVS9 A>G)

*This table includes all previously published reports of Christianson Syndrome with ESES. EEG, electroencephalogram; AEDs, anti-epileptic drugs; ESES, electrical status epilepticus during sleep; ID, intellectual disability; y, years; m, months; GTCS, generalized tonic-clonic seizures; TCS, tonic-clonic seizures; NK, unknown; VPA, valproic acid; CLB, clobazam; LEV, levetiracetam; CBZ, carbamazepine; ESM, ethosuximide; TPM, topiramate; LTG, lamotrigine; OXC, oxcarbazepine*.

* *negative behavioral impact*.

More than 80 genetic variants of *SLC9A6* have been reported to date. The most common pathogenic variants are protein-truncating mutations, such as frameshift or non-sense mutations and splicing mutations in transmembrane ion translocation domain (amino acid 25–533), which lead to partial or complete loss of NHE6 function (14). Pathogenic missense variants, small inframe deletions or non-sense mutations in the C-terminal cytoplasmic regulatory domain are relatively less common (1, 13). Genotype-phenotype correlations have not yet been established in CS. The c.1237-2 (IVS9) A>G variant is a canonical splice site mutation associated with exon 10 skipping, leading to a 38-amino-acid deletion (p.Leu381_Phe418del). Exon 10 in *SLC9A6* encodes the transmembrane ion translocation domain that interacts with the angiotensin II type 2 receptor (AGTR2) (10). Mutations in this region are associated with intellectual disability and epilepsy, although the underlying mechanisms remain to be investigated. Recently, arborization phenotypes were found to be rescued by the application of exogenous trophic factors (BDNF) across all type of *SLC9A6* mutation (20). Restraining over-acidification of endosomes and aberrant substance transportation or lysosomal degradation could also be potential therapeutic strategies for the treatment of CS patients with *SLC9A6* pathogenic variants.

## Conclusion

ESES observed early in the disease course is considered to be a significant feature of CS with the *SLC9A6* variants. Combined genetic analysis at both the DNA and RNA levels is necessary to confirm the pathogenicity of this variant and its role in the clinical diagnosis of CS.

## Data Availability Statement

The datasets presented in this article are not readily available due to ethical and privacy restrictions. Requests to access the datasets should be directed to the corresponding author.

## Ethics Statement

The studies involving human participants were reviewed and approved by Children's Hospital of Chongqing Medical University Ethics Committee. Written informed consent to participate in this study was provided by the participants' legal guardian/next of kin. Written informed consent was obtained from the minor(s)' legal guardian/next of kin for the publication of any potentially identifiable images or data included in this article.

## Author Contributions

XL: study conception and design, genetic testing, analysis of data, and drafting of manuscript. LX: genetic testing, analysis of data, and critical revision. ZF: acquisition of data, genetic testing, analysis of data, and critical revision. LJ: study conception and design, analysis of data, and critical revision. All authors contributed to the article and approved the submitted version.

## Conflict of Interest

The authors declare that the research was conducted in the absence of any commercial or financial relationships that could be construed as a potential conflict of interest.

## Publisher's Note

All claims expressed in this article are solely those of the authors and do not necessarily represent those of their affiliated organizations, or those of the publisher, the editors and the reviewers. Any product that may be evaluated in this article, or claim that may be made by its manufacturer, is not guaranteed or endorsed by the publisher.
